# Patient Compliance With Oral Health Care Advice: A Prospective Study of Older People With Memory Complaints

**DOI:** 10.1016/j.identj.2025.103900

**Published:** 2025-09-26

**Authors:** Sanne M. Pruntel, Arjan Vissink, Anita Visser

**Affiliations:** a Department of Oral and Maxillofacial Surgery, University Medical Center Groningen and University of Groningen, Groningen, The Netherlands; b Department of Gerodontology, Center for Dentistry and Oral Hygiene, University Medical Center Groningen and University of Groningen, Groningen, The Netherlands

**Keywords:** Memory complaints, Oral health advice, Oral health, Older people

## Abstract

**Background:**

Oral health is important for preserving oral function, general health, and overall well-being. However, obtaining and maintaining oral health is challenging for cases with memory complaints.

**Aim:**

To assess compliance of older people with memory complaints and oral health problems with given oral health care advice.

**Material and Methods:**

Consecutive patients visiting the memory clinic of the University Medical Center Groningen, the Netherlands, between May 2022 and October 2024 for the first time were asked whether they were willing to participate in a prospective study aimed at assessing the oral health and compliance with oral care advice. Exclusion criteria: not understanding Dutch, unable to follow instructions, or being incapacitated. After assessing the oral health and providing oral care advice, the compliance with the oral care advice was assessed after 6 weeks. If the given advice was not followed, another check was done 6 weeks later.

**Results:**

All 124 included patients needed oral health care advice. After 6 weeks, 114 of the patients (91.9%) had not followed the oral health care advice. At the recall 6 weeks later, 108/124 patients (87.1%) had still not followed the given advice.

**Conclusion:**

The compliance with oral care advice among older people with memory complaints and who need oral care is very low (12.9%).

## Introduction

Oral health is not only important for preserving oral functions (eg, chewing, speaking, swallowing), but also for general health and overall well-being.^1^ The importance of the link between general health, overall well-being and oral health is increasingly being recognized.^2^^,^^3^ Poor oral function can result from various factors, including hyposalivation, tooth loss, broken teeth, oral pain, dental caries, periodontal disease, candidiasis, etc.^4^^,^^5^ This, in turn, can lead to adverse health outcomes such as malnutrition, infections, inflammation, aspiration, poor quality of life, etc.^4^^,^^5^ Periodontal infections are, particular, considered to be a risk factor for systemic inflammation as it was shown that periodontal bacteria can enter the body. According to the literature, these infections are bidirectionally related to major comorbidities such as rheumatoid arthritis, diabetes mellitus, and cardiovascular disease.^3^^,^^6^^,^^7^

To prevent oral health issues, oral self-care as well as dental visits are *sine qua non* conditions.^8^ In older people, particularly frail older people with dementia, oral self-care is often poor and dental visits are frequently avoided due to severe (mental) health problems.^1^^,^^9^ Therefore, frail older people with dementia are at risk of oral health problems which, in turn, can result in general health issues and poor quality of life.^10^^,^^11^ This risk increases significantly when oral self-care and dental visits cease completely, and no additional oral care is provided by third parties such as informal carers.^10^

As written above, oral health and the prevention of oral health problems are important but, unfortunately, both are often overlooked in the management of patients with memory complaints.^12^^,^^13^ It is hypothesized that when oral screening is carried out at the onset of memory complaints and proper oral health advice is provided, severe oral health problems can be mitigated.^14^ This can, in turn, reduce their negative impact on general health and quality of life, thereby benefiting the patient.^14^

The effectiveness of proper oral health advice depends on two things: the quality of the recommendations and the patient’s cognitive and functional ability to understand and implement them.^15^ Most oral health professionals give advice on a daily basis and expect patients to follow it. However, they often do not thoroughly check whether this advice is indeed being followed or how effective it is in helping the patients to maintain oral health, particularly among older people with memory complaints. Additionally, the literature provides limited insight into the extent to which older people with memory complaints adhere to oral health care advice. Furthermore, there is little information on the factors that encourage or discourage such compliance.^1^ Adherence may be especially challenging in this population due to forgetfulness, reduced executive functioning, limited insight, and reduced support systems.^16^

A clear understanding of how older individuals with memory complaints adhere to oral health care advice is, therefore, essential to developing more effective, tailored interventions. Exploring the relationship between memory complaints, oral health, and adherence to oral health care advice could help oral healthcare professionals adjust their approaches and support this vulnerable group better. Therefore, the aim of this study was to assess the compliance of older people with memory complaints with oral care advice.

## Methods

### Study design and participant population

All consecutive home-dwelling patients visiting the memory clinic of the University Medical Center Groningen (the Netherlands) between May 2022 and October 2024 for the first time because of their memory complaints were asked whether they were willing to participate in a prospective study aimed at assessing the compliance with given (individually adjusted) oral care advice. Written informed consent was obtained from the participants and/or their carers. At the time of recruitment, the research team made an initial assessment of the participants’ capacity to provide informed consent. This assessment was based on their ability to understand the study-related information, engage in meaningful conversation, and make consistent, voluntary decisions. When their capacity was deemed insufficient, informed consent was also obtained from the informal carer. Exclusion criteria were: not understanding Dutch, unable to follow instructions, or being incapacitated.

This study was approved by the Institutional Review Board of the University Medical Centre Groningen (the Netherlands research registration number 202000809).

### Procedure

During their visit to the memory clinic, all the participants’ demographic characteristics (age, gender, and smoking) and oral health were assessed (see below for details). The participants and their carers were then informed about the oral-(self) care requirements. Individual oral health care advice was provided, and their general dental practices were also informed (see below for details). After 6 weeks, the participants and/or their informal carers were phoned and asked whether the given oral health care advice had been followed. If it had not been followed, the advice was repeated, and the participants were asked to specify the reasons for noncompliance. A second follow-up assessment was then conducted after an additional 6 weeks to determine whether the regiven advice had been followed. Participants were considered to be compliant if they had executed the instructed advice fully.

### Oral health assessment

The need for oral (health) care was determined following an oral health assessment. This oral health assessment included the following: the presence of deep carious lesions (ICDAS 4 and above),^17^ gingivitis (bleeding on probing), periodontal pockets (depth in mm), chewing problems (inability to eat properly), broken teeth, mobile teeth, problems with complete and partial dentures, mucosal pathology, and bone pathology. Caries and bone pathology were assessed using radiographs. If the oral health was good, no advice was needed. If there were oral health problems, the participants were shown and instructed on good oral self-care (brushing, rinsing, interdental cleaning, dietary advice, etc.) and were advised to (re)visit and consult their general dental practice for specific treatment such as tooth restoration or periodontal treatment, etc.

These recommendations were given orally to the participants and/or carers, and were also handed over printed on paper. If the participant and/or their carer agreed, information about their current oral health status and the radiograph was sent to their general dental practice.

### Statistical analyses

All statistical analyses were performed using SPSS IBM Statistics version 23.0 (SPSS). A chi-squared test for independence was used to compare the frequency of dental visits between the participants with complete dentures and those with natural dentition. Data normality was assessed with the Shapiro–Wilk test. Descriptive statistics were used to provide an overview of the demographic characteristics and oral health. A multivariable logistic regression analysis was conducted to evaluate the association between specific oral health care advice and patient compliance at six and 12 weeks. Compliance was treated as the dependent binary variable (yes/no) and each advice item was included as an independent variable along with the relevant demographic covariates, such as age, smoking, and natural dentition. Odds ratios with corresponding 95% confidence intervals and *P* values were calculated. *P* < .05 was considered statistically significant.

## Results

### Participants

Table 1 shows the demographics of the included participants. During the inclusion phase, 187 patients were asked to participate, but of those, 124 participants were included. A total of 63 eligible patients did not participate (Table 2).

**Table 1:** Participant characteristics.

	Participants (*n* = 124)
Gender: female, *n* (%)	53 (42.7)
Age in years, mean (SD)	73.9 (7.2)
Smoking	69 (55.6)
Natural dentition, *n* (%)	90 (72.6)
Number of teeth, mean (SD)	15.7 (10.9)
A maxillary partial denture, *n* (%)	8 (6.4)
A mandibular partial denture, *n* (%)	10 (8.0)
Edentulous, *n* (%)	32 (25.8)
A complete maxillary denture, *n* (%)	42 (33.9)
A complete mandibular denture, *n* (%)	32 (25.8)
Implant-retained overdenture, *n* (%)	14 (11.3)
Natural dentition and still visits their general dental practice regularly, *n* (%)	78 (86.6)
Edentulous and still visits their general dental practice regularly, *n* (%)	2 (5.9)

**Table 2:** Reasons for patients not participating.

Reason	Number of patients (*n* = 63)
Did not want any additional hospital visits	31 (49.2%)
Too anxious about memory complaints	24 (38.1%)
Did not want to undergo radiation	4 (6.3%)
Bedridden	4 (6.3%)

### Oral health

Most participants (72%) still had remaining teeth. Among the participants with complete dentures (*n* = 34; 27.4%), only a few (*n* = 2; 5.9%) still visited their general dental practice on a regular basis, which is significantly lower compared to those participants with their own remaining teeth (*n* = 78; 86.6%) (Table 1).

All 124 of the included participants needed oral health care and received oral health advice accordingly (Tables 3 and 4). Just over half of the participants smoked. Of the participants who still had some of their own teeth, most had one or more deep carious lesions and/or deep periodontal pockets (Table 3). A wide range of oral health care advice was given to the participants (Table 4). Most were advised to start or improve their oral hygiene, in particular, on how to perform interdental cleaning. More than one type of oral health care advice was given to 30 (24.2%) of the participants, eg, on food intake and the need to visit a dentist.

**Table 3:** Observed oral health problems.

Oral health problems	Participants *n* (%) (total number [*n*] of participants = 124)
Deep carious lesions (ICDAS 4 and up)	68 (54.8)
Periodontal pockets ≥6 mm	72 (58.1)
Gingivitis (swollen and red gums)	76 (61.3)
Chewing problems	49 (39.5)
Mobile teeth	18 (14.5)
Complete and partial denture problems:	29 (23.4)
– Maxillary denture	13 (10.5)
– Mandibular denture	4 (3.2)
– Both dentures	12 (9.7)
Mucosal pathology	23 (18.5)
Bone pathology:	21 (16.9)
– Periapical radiolucencies	19 (15.3)
– Periapical opaque lucencies	2 (1.6)

**Table 4:** Advice during the oral assessments.

Advice	Participants *n* (%) (total number [*n*] of participants = 124)
Make an appointment with an oral health care professional for treatment and further consultation	39 (31.4)
Brushing instructions for natural teeth	4 (3.2)
Brushing instructions for denture	36 (29.0)
Dental prophylaxis (the use of toothpaste or toothpaste with fluoride at all)	4 (3.2)
Adjust the brushing frequency	33 (26.6)
Advice on interdental cleaning	57 (46.0)
Advice to start brushing with an electric toothbrush	32 (25.8)
Advice to limit eating occasions to 7 per day	25 (20.6)
Advice to reduce the number of sugary occasions	60 (48.4)

### Recall

When the participants and/or informal carers were phoned after 6 weeks for the first time, 114 of the participants (91.9%) had not followed the given advice. The participants were given the respective advice again and scheduled for another assessment 6 weeks later to determine whether the advice had been followed (Table 5). During this second assessment, a total of 108 out of the 124 participants (87.1%) had still not followed the given oral health care advice.

**Table 5:** Number of participants who followed or did not follow the oral health care advice.

Responders; *n* (%), total number of participants 124	After first 6 wk; *n* (%)	After 12 wk (second call after another 6 wk); *n* (%)	In total; *n* (%)
Followed the advice	10 (8.1)	16 (12.9)	16 (12.9)
Did not follow the advice	114 (91.9)	108 (87.1)	108 (87.1)
Total	124	124	124

The most mentioned reasons for not following the advice varied (Table 6): no time yet, on my to-do list, no money, not a priority, expected the dentist to call, thought the research team would call, not reminded of the given advice, no transport, and no reason. The most commonly reported reason was: not having had the time yet. Furthermore, 15 participants stated that maintaining good oral health was not a priority for them, even after a second call from the researchers during which they were reminded that they needed oral health care. Thirteen of those participants and their carers could not even remember the given advice.

**Table 6:** Reasons given by participants at the six and 12-week follow-ups for not following the oral health advice.

Reasons for not following the advice	Participants who did not follow the advice; *n* (total *n* = 114) after 6 wk (%)	Participants who did not follow the advice; *n* (total *n* = 108), after 12 wk (%)
No time yet	40 (35.1)	37 (34.3)
Not on my to-do list	7 (6.1)	5 (4.6)
Not a priority	16 (14.0)	15 (13.9)
Expected their dentist to call	12 (10.5)	12 (11.1)
Thought researchers would call for an appointment	2 (1.8)	2 (1.9)
Not reminded of given advice	13 (11.4)	13 (12.0)
No transport	8 (7.0)	8 (7.4)
No reason	16 (14.1)	16 (14.8)
Total	114	108

### Association between age, smoking, natural dentition, and compliance

Table 7 gives an overview of the associations between age, smoking, natural dentition, and compliance after 6 and 12 weeks.

As can be seen in Table 7, age, smoking, and natural dentition did not seem to be associated with compliance with any of the given advice.

**Table 7:** Associations between age, smoking, natural dentition, and compliance after 6 and 12 weeks.

Optional compliance predictor	Estimate (compliance 6 wk)	OR (95% CI) (6 wk)	*P* value (6 wk)	Estimate (compliance 12 wk)	OR (95% CI) (12 wk)	*P* value (12 wk)
Age	–0.081	0.92 (0.81-1.04)	.195	–0.008	0.99 (0.91-1.08)	.848
Smoking	2.594	13.41 (0.85-210.57)	.065	0.034	1.03 (0.30-3.55)	.956
Natural dentition	–0.960	0.38 (0.01-14.90)	.607	–0.978	0.38 (0.03-4.72)	.448
Make an appointment with an oral health care professional for treatment and further consultation	2.222	9.19 (0.97-87.45)	.053	0.461	1.59 (0.44-5.71)	.481
Denture brushing instructions	0.981	2.67 (0.12-57.48)	.531	–0.227	0.80 (0.15-4.25)	.791
Dental prophylaxis	–19.907	0.00 (0.00-inf)*	.999	–23.180	0.00 (0.00-inf)*	.999
Advice on interdental cleaning	–1.264	0.28 (0.02-3.25)	.310	–0.202	0.82 (0.22-3.03)	.763
Natural teeth brushing instructions	5.463	235.75 (1.98-28,104.79)	.025	1.851	6.37 (0.93-43.54)	.059
Adjust the brushing frequency	–226.788	0.00 (0.00-inf)*	1.000	–0.716	0.49 (0.11-2.13)	.341
Advice to limit eating occasions to 7 per day	0.165	1.18 (0.04-35.99)	.925	0.776	2.17 (0.18-26.41)	.543
Advice to reduce the number of sugary occasions	–2.201	0.11 (0.00-3.15)	.198	–1.722	0.18 (0.02-2.07)	.169
Advice to start brushing with an electric toothbrush	–3.238	0.04 (0.00-0.85)	.039	–1.288	0.28 (0.06-1.17)	.081

CI, confidence interval; OR, odds ratio.

⁎ An OR of 0.00 with an infinite confidence interval suggests either a lack of events in one group or model instability.

These findings suggest that neither age nor smoking status had a statistically significant influence on the participants’ compliance with oral healthcare advice at either time point.

Logistic regression analysis revealed that two specific pieces of advice were significantly associated with patient compliance at 6 weeks. These were: the ‘Natural teeth brushing instructions’ and ‘Advice to start brushing with an electric toothbrush’. The patients who received either of these pieces of advice were more likely to comply. In contrast, the remaining oral health care advice was not significantly associated with compliance, neither after 6 weeks nor after 12 weeks.

## Discussion

The aim of this prospective study was to assess the compliance of older people with memory complaints who were given oral (health) care advice. All the participants needed such advice. Although all the participants received one or more oral health care advice, and this advice was also sent to their general dental practitioner when given permission to do so, most of them (87.1%) did not follow the oral health advice, even after 2 telephone calls aimed to remind them about the given advice.

This is, however, not remarkable as studies have shown that people with memory issues, such as dementia, often struggle to follow instructions due to cognitive impairments that affect memory, attention, and comprehension. For example, dementia can affect the ability to process and retain new information, making it difficult for patients to remember and follow instructions.^18^^,^^19^

Next, the high need for oral care that was found in this study is not new either: older people with memory complaints often have oral health care problems.^20^^,^^21^ However, to the best of our knowledge, the compliance with oral health care advice by patients with memory complaints has never been described in literature. Although most of the participants and their carers recognized the importance of the given oral health care advice, 87.1% of them had not taken action within 3 months after the oral health advice was given. Just over 10% of the older people with memory complaints could not even recall that oral health advice had been given, neither could their carers, even when the advice was given both orally and printed on paper. Perhaps their carers had some cognitive issues as well, or had to organize so many other things that oral health had become of less importance.

Patients with memory complaints often struggle with maintaining good oral health, making compliance with oral care advice particularly challenging.^1^ However, it is notable that even when advice was given both verbally and in printed form, and their carers are also informed, a significant proportion still did not remember the given advice. Some of them said they did not follow the advice because of not having time to take action. This explanation is striking as many of these people are no longer working and, theoretically, have plenty of time for self-care. This suggests that the problem is not a lack of time, but rather an inability to organize and perform oral care routines independently. This difficulty may indicate a need for additional support from healthcare professionals, which was also indicated by the participants. Some of them expected their dentist to remind them of the necessary oral care actions.

The reliance of the participants on external reminders and the fact that the oral health professionals did not actively provide such a service also indicates that this vulnerable group needs help with organizing care. Without a structured follow-up, essential oral health measures may be neglected, leading to potential deterioration in both oral and general health. In this respect, professional involvement in oral health care planning may be necessary to improve compliance. Regular active follow-up by dental professionals, integration of oral care into routine memory care programmes, cooperation with medical doctors, and structured reminders by healthcare providers could help to bridge this gap. Close cooperation and communication, maybe with the aid of a case manager, could help people with memory complaints to get the care they need. In the Netherlands, oral health care and general healthcare are organized separately.^22^ Memory clinics usually do not have standard referral protocols for oral health assessments.^23^ However, patients often come into contact with case managers or social workers who could facilitate such referrals in an integrated care setting.^24^

These above-mentioned findings have implications not only for individual care strategies but also for health policy more broadly. Despite the well-documented links between oral health and general well-being, oral health is still not fully integrated into care pathways for older adults with memory complaints. The results suggest that policymakers and care systems should consider to include oral health screening and oral health care interventions in routine multidisciplinary care for older adults with memory complaints. This could prevent severe oral health issues, which in turn can cause general health issues. From a health policy perspective, several concrete steps can be taken. Routine oral health assessments should preferably be incorporated into geriatric assessments, and oral health education should form part of the training of all healthcare professionals, especially those working with older people. Next, oral healthcare professionals should be part of geriatric teams and being involved in elderly care. Also, the communication between medical and dental professionals should become normal and efficient to ensure patients get the dental care they need. Furthermore, all (dental) care professionals should place emphasis on prevention, particularly when signs of cognitive complaints or frailty begin to appear, as the risk of poor oral health will increase when cognitive decline becomes apparent. And last, care professionals and policy makers should keep in mind that it would be wise to involve informal carers as well by informing them on the importance of daily oral hygiene and encourage them to maintain access to professional care. Informal carers play a crucial role in supporting oral health, but they can only fulfil this role if they are intrinsically motivated and instructed, which depends on their understanding of the importance of oral health. To this end, governments should invest in accessible educational resources, such as high-quality websites and public information campaigns. Thus, in practical terms, improvements to the oral healthcare of older adults with memory complaints could involve simple yet effective measures. For instance, carers could be taught to use checklists for daily oral hygiene routines or to implement visual prompts in the bathroom, such as illustrated instructions or timers. Oral healthcare professionals could provide short instructional videos or demonstrations during home visits, focusing not only on patients, but on carers as well. Carers should be encouraged and supported to observe changes in the mouth, such as sores, pain, or broken teeth, and to report these early. Empowering carers in this way requires not only information but also validation of their role as partners in care. Local community initiatives, such as carer cafés or training sessions organized by dental professionals, could strengthen the vital link between oral health and informal care.

Implementing oral care in general healthcare programmes for older people could prevent oral health issues. This might also be beneficial for people at earlier stages in life. Recent literature shows significant associations between oral health and cognitive functions such as learning and memory, complex attention, and executive function.^25^, ^26^, ^27^ In addition, a recent study showed that oral health interventions play a positive role in maintaining cognitive levels in people with dementia.^28^

In the context of dental care, older people with a natural dentition tend to use dental services more often than edentulous older people. The finding is almost in line with the Bakker et al^9^ findings, where 67% of the older people who had natural dentition still visited the dentist.

### Strengths and limitations

The strength of this study is that it is the first to examine oral health assessments and compliance with oral health advice among older people with memory complaints in a memory clinic setting. However, a limitation is the lack of a control group, such as a comparison group of older people, cognitively healthy individuals who received comparable oral health advice. Thus, generalization of the results should be performed with caution, eg, is the noncompliance to the oral health care advice due to memory complaints or other factors. Another limitation of this study is its reliance on self- or proxy-reported data regarding compliance. Due to the cognitive status of some participants, there is a possibility that recall or reporting bias was introduced, which could have affected the accuracy of the responses. However, all the included participants had informal carers who were also informed about the advice, and they could have helped with following the advice, which they did not. Noteworthy is that sometimes their carers did not understand either, even when the advice was given orally and in writing. The question is whether their carers could also have had cognitive problems.

## Conclusion

Among older people with memory complaints and who are in need of oral care, the compliance to take action in line with given oral health care advice is low, although their need for oral care is high. This highlights the importance of developing tailored strategies to overcome the fact that these older people often do not follow instructions or are unable to follow the given advice. An active approach, such as actively arranging an appointment with dental care professionals, more frequent visits, and home visits, might be beneficial to maintain oral care and thereby oral health in these frail older people. Further interventions, such as involving case managers, using digital reminders for patients and carers, and establishing community-based dental outreach programmes, could enhance adherence further and ensure that oral health needs are met in a timely and sustainable manner.

## Author contributions

All listed authors (Sanne M. Pruntel, Arjan Vissink, and Anita Visser) have made substantial contributions to (1) the conception or design of the work or the acquisition, analysis or interpretation of data for the work; (2) drafting the work or revising it critically for important intellectual content; (3) giving final approval of the version to be published; and (4) agreeing to be accountable for all aspects of the work, ensuring that questions related to the accuracy or integrity of any part of the work are appropriately investigated and resolved. All authors have read and agreed to the published version of the manuscript.

## Conflict of interest

S.M. Pruntel is financially supported by a research grant from Health-Holland, Top Sector Life Sciences & Health TKI-LSH-T2021-ORANGE-Force; https://www.health-holland.com/nl/public-private-partnerships/orange-health-nl. Other authors declare that they have no known competing financial interests.

## References

[1] Delwel S., Binnekade T.T., Perez RSGM, Hertogh CMPM, Scherder E.J.A., Lobbezoo F. (2018). Oral hygiene and oral health in older people with dementia: a comprehensive review with focus on oral soft tissues. Clin Oral Investig.

[2] Paul O., Arora P., Mayer M., Chatterjee S. (2021). Inflammation in periodontal disease: possible link to vascular disease. Front Physiol.

[3] Pruntel S.M., van Munster B.C., de Vries J.J., Vissink A., Visser A. (2024). Oral health as a risk factor for Alzheimer disease. J Prev Alzheimer’s Dis.

[4] Offenbacher S., Barros S.P., Altarawneh S., Beck J.D., Loewy ZG. (2012). Impact of tooth loss on oral and systemic health. Gen Dent.

[5] Azzolino D., Passarelli P.C., De Angelis P., Piccirillo G.B., D’addona A., Cesari M. (2019). Poor oral health as a determinant of malnutrition and sarcopenia. Nutrients.

[6] Brewer R.C., Lanz T V, Hale C.R. (2023). Oral mucosal breaks trigger anti-citrullinated bacterial and human protein antibody responses in rheumatoid arthritis. Sci Transl Med.

[7] Lamster IB., Lalla E., Borgnakke WS., Taylor GW. (2008). The relationship between oral health and diabetes mellitus. J Am Dent Assoc.

[8] Bakker M.H., Vissink A., Spoorenberg S.L.W., Wynia K., Visser A. (2020). Self-reported oral health problems and the ability to organize dental care of community-dwelling elderly aged ≥75 years. BMC Oral Health.

[9] Bakker M.H., Vissink A., Raghoebar G.M., Peters L.L., Visser A. (2021). General health, healthcare costs and dental care use of elderly with a natural dentition, implant-retained overdenture or conventional denture: an 8-year cohort of Dutch elderly (aged 75 and over). BMC Geriatr.

[10] Ginó S., Mendes T., Maroco J. (2010). Memory complaints are frequent but qualitatively different in young and elderly healthy people. Gerontology.

[11] Skallevold H.E., Rokaya N., Wongsirichat N., Rokaya D. (2023). Importance of oral health in mental health disorders: an updated review. J Oral Biol Craniofac Res.

[12] Lopez-Jornet P., Lavella C.Z., Lopez E.P.F., Tvarijonaviciute A. (2021). Oral health status in older people with dementia: a case—control study. J Clin Med.

[13] Barker A., Prior J., Jones R. (1995). Memory complaint in attenders at a self-referral memory clinic: the role of cognitive factors, affective symptoms and personality. Int J Geriatr Psychiatry.

[14] Taiwo O.O., Panas R. (2018). Roles of community pharmacists in improving oral health awareness in Plateau State, Northern Nigeria. Int Dent J.

[15] Soldani F.A., Lamont T., Jones K. (2018). One-to-one oral hygiene advice provided in a dental setting for oral health. Cochrane Database Syst Rev.

[16] Dolansky M.A., Hawkins M.A.W., Schaefer J.T. (2016). Association between poorer cognitive function and reduced objectively monitored medication adherence in patients with heart failure. Circ Heart Fail.

[17] Gupta M., Gugnani N., Pandit I. (2011). International caries detection and assessment system (ICDAS): a new concept. Int J Clin Pediatr Dent.

[18] Banovic S., Zunic L., Sinanovic O. (2018). Communication difficulties as a result of dementia. Mater Socio Med.

[19] Cipriani G., Danti S., Picchi L., Nuti A., Di Fiorino M. (2020). Daily functioning and dementia. Dement Neuropsychol.

[20] Jiang Z., Liu X., Lü Y. (2022). Unhealthy oral status contributes to the older patients with cognitive frailty: an analysis based on a 5-year database. BMC Geriatr.

[21] Li A., Chen Y., Visser A., Marks L.A.M., Tjakkes GHE. (2022). Combined association of cognitive impairment and poor oral health on mortality risk in older adults: results from the NHANES with 15 years of follow-up. J Periodontol.

[22] Niesten D., Gerritsen A.E., Leve V. (2021). Barriers and Facilitators to integrate oral health care for older adults in general (basic) care in East Netherlands. Part 2 Functional Integration. Gerodontology.

[23] Schaper S., Meyer-Rötz S., Bartels C. (2021). Dental care of patients with dementia: a survey on practice equipment, training, and dental treatment. Front Oral Health.

[24] Lian T., Kutzer K., Gautam D. (2021). Factors associated with patients’ connection to referred social needs resources at a federally qualified health center. J Prim Care Community Health.

[25] Nangle M.R., Riches J., Grainger S.A., Manchery N., Sachdev P.S., Henry JD. (2019). Oral health and cognitive function in older adults: a systematic review. Gerontology.

[26] Stewart R., Weyant R.J., Garcia M.E. (2013). Adverse oral health and cognitive decline: the health, aging and body composition study. J Am Geriatr Soc.

[27] Manchery N., Henry J.D., Lam B.C.P. (2022). Memory decline in older individuals predicts an objective indicator of oral health: findings from the Sydney Memory and Ageing Study. BMC Oral Health.

[28] Guo H., Wang Z., Chu C.H., Chan A.K.Y., Lo E.C.M., Jiang CM. (2024). Effects of oral health interventions on cognition of people with dementia: a systematic review with meta-analysis. BMC Oral Health.

